# Predicting the Response to Intravenous Immunoglobulins in an Animal Model of Chronic Neuritis

**DOI:** 10.1371/journal.pone.0164099

**Published:** 2016-10-06

**Authors:** Gerd Meyer zu Horste, Steffen Cordes, Johannes Pfaff, Christian Mathys, Anne K. Mausberg, Martin Bendszus, Mirko Pham, Hans-Peter Hartung, Bernd C. Kieseier

**Affiliations:** 1 Department of Neurology, Heinrich-Heine-University, Medical Faculty, Düsseldorf, Germany; 2 Department of Neurology, University Hospital Münster, Münster, Germany; 3 Department of Neuroradiology, Heidelberg University Hospital, Heidelberg, Germany; 4 Institute of Neuroradiology, Heinrich-Heine-University, Medical Faculty, Düsseldorf, Germany; Hannover Medical School, GERMANY

## Abstract

Chronic inflammatory demyelinating polyradiculoneuropathy (CIDP) is a disabling autoimmune disorder of the peripheral nervous system (PNS). Intravenous immunoglobulins (IVIg) are effective in CIDP, but the treatment response varies greatly between individual patients. Understanding this interindividual variability and predicting the response to IVIg constitute major clinical challenges in CIDP. We previously established intercellular adhesion molecule (ICAM)-1 deficient non-obese diabetic (NOD) mice as a novel animal model of CIDP. Here, we demonstrate that similar to human CIDP patients, ICAM-1 deficient NOD mice respond to IVIg treatment by clinical and histological measures. Nerve magnetic resonance imaging and histology demonstrated that IVIg ameliorates abnormalities preferentially in distal parts of the sciatic nerve branches. The IVIg treatment response also featured great heterogeneity allowing us to identify IVIg responders and non-responders. An increased production of interleukin (IL)-17 positively predicted IVIg treatment responses. In human sural nerve biopsy sections, high numbers of IL-17 producing cells were associated with younger age and shorter disease duration. Thus, our novel animal model can be utilized to identify prognostic markers of treatment responses in chronic inflammatory neuropathies and we identify IL-17 production as one potential such prognostic marker.

## Introduction

Inflammatory polyneuropathies constitute disabling autoimmune mediated disorders of the peripheral nervous system (PNS). Acute and chronic variants have been described. The acute Guillain-Barré syndrome (GBS) features rapid onset monophasic inflammation of the PNS [1, 2] and experimental autoimmune neuritis (EAN) serves as an animal model of its demyelinating variant [3]. Chronic inflammatory demyelinating polyradiculoneuropathy (CIDP)–the most common chronic inflammatory neuropathy–presents with slowly progressive or relapsing remitting sensory and motor impairments due to immune cell infiltration of peripheral nerves [4, 5]. Histologically, infiltrates of T lymphocytes and macrophages can be demonstrated in the PNS of CIDP patients [6]. Glucocorticoids, plasma exchange and intravenous immunoglobulins (IVIg) constitute established treatment options in CIDP, but do not benefit all patients [7]. Significant and chronic disability is therefore frequent in CIDP [5]. Among the available treatment options, IVIg features the most advantageous risk-to-benefit ratio and has long-term positive effects [8], but its efficacy varies greatly between individual patients and its mechanism of action in inflammatory neuropathies remains poorly defined. Identifying prognostic markers of treatment responses in IVIg is clinically highly relevant.

Different modes of action have been described including effects on autoantibodies, Fc immunoglobulin fragment receptors and on pro-inflammatory cytokines (reviewed in [9]). Evidence supports that the various IVIg effects are mediated by its Fc portion as Fc fragment preparations were sufficient to ameliorate rat EAN [10, 11]. Effects of IVIg on the expression of the anti-inflammatory immunoglobulin receptor FcγRIIB on B cells has been reported in CIDP patients [12]. IVIg ameliorates the acute EAN rat model [13]. The relevance of this finding for CIDP is unknown and IVIg treatment has not been tested in chronic inflammatory neuropathy animal models, which were only recently introduced. Such animal models of chronic inflammatory neuropathies could help to extend our understanding of the IVIg effect [14].

We and others have previously reported, that mice of the autoimmune-prone non-obese diabetic (NOD) strain with deficiency in the costimulatory molecules B7-2 [15] and intercellular adhesion molecule (ICAM)-1 [16] spontaneously develop chronic inflammation and demyelination of peripheral nerves and constitute potential animal models of CIDP.

We here used ICAM-1^-/-^NOD mice to further clarify IVIg effects in this chronic inflammatory neuropathy model and identified production of interleukin (IL)-17 as one potential prognostic marker predicting a beneficial effect of IVIg treatment. In sural nerve biopsy sections of human CIDP patients, IL-17 producing cells were more prevalent in young patients with shorter disease duration.

## Material and Methods

### Animals and Phenotyping

Animal experimentation was approved by the responsible state authorities (LANUV NRW) under the approval reference number AZ 84–02.04.2011.A128. All animals were maintained under specific pathogen free conditions. ICAM-1^-/-^ mice on C57/BL6 background [17] were backcrossed to NOD background (MHC haplotype H-2^g7^, Bomholtgard, Denmark) for 8 generations as previously described [18] and homozygous ICAM-1^-/-^NOD mice were further inbred. Homozygozity was confirmed by routine PCR from tail biopsies in randomly chosen animals as previously described [17]. ICAM-1^-/-^NOD mice were weekly analyzed for clinical signs of neuropathy for the duration of the treatment in a blinded fashion by the same investigator (S.C.). A modified EAN score [19] was applied: 0 no impairments, 1 reduced tone of the tail, 2 limp tail, 3 absent righting reflex, 4 gait ataxia, 5 mild paraparesis, 6 moderate paraparesis, 7 severe paraparesis or paraplegia, 8 tetraparesis, 9 moribund, 10 death due to neuropathy.

### IVIg Treatment and Study Design

A cohort of 60 female ICAM-1^-/-^NOD mice with an average age of 227 days (median 229 days, range 169–302 days) and an average clinical score of 0.69 (median 0.00, range 0–4) was assessed by sciatic nerve electrophysiology (see below). Two littermate animals with clinical scores greater than 4 were excluded. Animals were then randomized stratified first by nerve conduction velocities and second by clinical score into three cohorts (n = 20 each). Cohorts either received intraperitoneal injections of 10 mg human albumin (100 μl of 100 mg/ml, company CSL Behring) three times per week (placebo group) or 10 mg human IVIg preparation (100 μl of 100 mg/ml solution Kiovig, Baxter) three times per week (IVIg 3x group) or once per week (IVIg 1x group) for nine weeks. The average clinical score in the last three weeks of treatment was substracted from the average score in the first three weeks. Responders were defined as animals with ≤ 1 score point increase or reduction of the clinical score during treatment. Non-responders were defined as <1 score increase. Blood was taken from the animals before treatment initiation by tail vein bleeding and intracellular cytokine staining of blood lymphocytes was performed (see below). At the end of the study, sciatic nerve electrophysiology was performed and animals were sacrifized by cervical dislocation. Sciatic nerves were dissected and one nerve was stored at -80°C for expression analysis and one fixed in 4% paraformaldehyde solution for histology. Spleens were dissected for immediate cytokine staining and proliferation analysis.

### Electrophysiology

Mouse sciatic nerve conduction properties were determined before treatment initiation and at the end of the experiment as previously described [20]. Briefly, mice were anaesthetized using brief inhalation narcosis with isofluran (Baxter), while constant body temperature was maintained using a heating plate connected to a rectal temperature sensor. Recording was performed using a portable human electrophysiology device adapted to the setting (Dantec). Two recording electrodes were inserted into the small foot muscles to assess motor response. Two monopolar stimulating electrodes were placed dorsal of the ankle and at the sciatic notch enclosing the sciatic nerve for distal and proximal stimulation, respectively. Stimulation was performed with increasing current until supramaximal stimulation was achieved. Maximum compound muscle action potential (CMAP) amplitude voltage (mV) was recorded. Nerve conduction velocity (NCV, m/s) was calculated as the quotient between the distance and the difference of motor latencies between proximal and distal stimulation. Average values were calculated from two independent recordings per animal.

### Histology

Fixed sciatic nerves from randomly chosen animals from the placebo (n = 8) and IVIg 3x group (n = 10) were cut into proximal, mid and distal pieces and paraffin embedded. Nerves were cut into 7 μm sections on a standard microtome and haematoxylin-eosin (HE) stained following standard protocols and dehydrated and mounted in xylene based medium (Merck). Entire sciatic nerve sections at proximal, mid and distal level were photographed in parallel at 40x magnification using a standard microscope (Zeiss). Photographs were merged and converted to binary (threshold 80) using Photohop CS3 (Adobe). The number of cell nuclei was automatically quantified using the Analyze Particle function (size = 50–400 circularity = 0.40–1.00) of ImageJ (v1.45, NIH). The endoneural area was measured and the nuclear density (nuclei / mm^2^) was calculated for each section image. To verify automatic quantification, nuclei were manually counted in six randomly selected images using the cell counter plug-in of ImageJ. Values closely correlated with automatic values. Luxol Fast Blue (LFB) staining was performed by deparaffinizing sciatic nerve sections, staining with 0.1% Luxol Blue for 18 hours at 57°C, differentiating with 0.1% lithium carbonate for one minute and mounting the sections. Photos of the stained sections were taken, converted to grayscale and the relative darkness (0 white, 255 black) of the endoneural area was quantified with Image J.

### Cell Preparation and Flow Cytometry

Intracellular cytokine staining was performed using blood mononuclear cells at the beginning and splenocytes after completion of the study. Proliferation analyses were performed using splenocytes at the end of the study.

Spleens were passed through a 40 μm cell strainer followed by erythrocyte lysis (both BD Biosciences). For proliferation analysis, splenocytes were cultured in the presence of syngenic mouse sciatic nerve homogenisate (50 mg/ml) or stimulated using soluble antibody against CD3 (1μg/ml, clone 145-2C11, from BD Pharmingen) and CD28 (0.5 μg/ml, clone PV-1, from Abcam). 2x10^5^ splenocytes were maintained in 96-well plates for 96 hours at 37°C in a humidified CO_2_ incubator. ^3^H-Thymidin (Hartmann Analytic) was added for the last 24 hours and proliferation was assessed in quadruplicate wells by measuring ^3^H-Thymidine incorporation. Stimulatory indices were calculated by dividing counts per minute (CPM) of each well by the average CPM of non-stimulated wells.

Blood taken by tail vein bleeding was collected in 75 μl hematocrit capillaries (Radiometer Clinical Aps) and transferred into 1ml of PBS/1%FCS/10mM EDTA solution followed by erythrocyte lysis. For intracellular cytokine staining, blood or spleen cells were cultured for 4 hours in 96-well plates at 37°C in a humidified CO_2_ incubator in the presence of PMA (10 ng/ml), ionomycin (1 μg/ml) and BD Golgistop. Cells were subsequently stained for cell surface CD4 (clone L3T4), CD3 (clone 145-2C11) and intracellular IL-17 (clone TC11-18H10) using the intracellular cytokine staining buffer set following the manufacturer’s protocol (all from BD Biosciences). Flow cytometry was performed using a FACSCanto II flow cytometer (BD Biosciences).

### Magentic Resonance Imaging

Magnetic resonance imaging (MRI) in mice was performed during inhalation narcosis with isofluran (4% in O_2_) using a 9.4T magnetic resonance (MR) scanner (Biospec 94/20, BRUKER, Ettlingen). A circular polarized cryoprobe was used in Tx/Rx setting. We acquired T2w images using a RARE (TR/TE: 2859/42ms, matrix: 320 x 320, pixel spacing: 0.0625 x 0.0625mm, slice thickness: 0.3mm, slices: 20, time: 13min 20sec) based sequence. Sciatic nerves were segmented and the normalized T2 signal was measured using ImageJ (v1.47, NIH) by defining the intensity of surrounding muscle tissue as 1.0 and calculating the relative T2 signal intensity of the sciatic nerve.

### Data Acquisition and Analysis

All flow cytometry data were analyzed using FlowJo software (v7.2.5 TreeStar). Data were statistically analyzed using GraphPadPrism 5.0 (GraphPad Software). The Wilcoxon-Mann-Whitney and Student’s t-test for unrelated samples were used to test for statistically significant differences of non-Gaussian and Gaussian distributed data, respectively. Differences were considered significant at p-values < 0.05.

## Results

### IVIg Treatment Ameliorates Chronic Inflammatory Neuropathy in ICAM-1^-/-^NOD Mice

We had previously established ICAM-1^-/-^NOD mice as a novel animal model of human CIDP [16]. We now asked whether this model could also help to improve our understanding of CIDP treatments and we therefore tested whether IVIg are effective in ICAM-1^-/-^NOD mice. We randomized a large cohort (n = 60) of pre-symptomatic and mildly affected ICAM-1^-/-^NOD mice (mean clinical score 0.59, mean age 7.6 months) into three matched groups (n = 20 each) stratified first to their NCV and second to their clinical score to receive intraperitoneal injections of either once weekly IVIg (IVIg 1x; mean score 0.67, mean age 7.4 months), or three times weekly IVIg (IVIg 3x; mean score 0.53, mean age 7.4 months) or three times weekly albumin (placebo; mean score 0.56, mean age 7.9 months). The randomized groups did not differ before treatment (S1 Table). Cytokine production by peripheral blood CD3+ T cells was quantified by intracellular cytokine staining before the start of IVIg treatment (see below).

**Fig 1:**
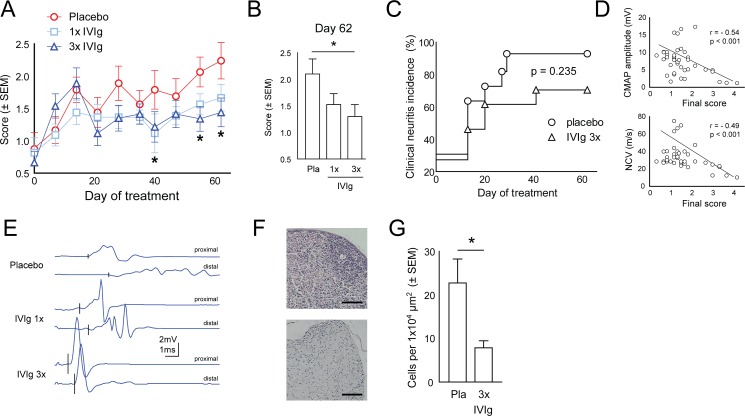
IVIg ameliorates chronic neuritis in ICAM-1^-/-^NOD mice. (A) We randomized a cohort (n = 60) of pre-symptomatic ICAM-1^-/-^NOD mice into three score-matched groups (n = 20 each) to receive either once weekly IVIg (IVIg 1x), three times weekly IVIg (IVIg 3x) or three times weekly albumin (placebo) treatment. The treatment was performed for 9 weeks in all animals and clinical scoring was performed weekly. (B) The average score at the end of the observation period in the placebo (pla) and IVIg treatment groups is shown. (C) The proportion of mice with clinically detectable impairment was quantified during treatment. ns not significant. (D) The compound muscle action potential (CMAP) amplitude and nerve conduction velocity (NCV) was measured after 9 weeks of treatment and correlated with the clinical score on the same date. (E) Sciatic nerve electrophysiology was performed by stimulating the sciatic nerve with needle electrodes at the gluteal (proximal) and ankle (distal) level and recording CMAPs with a needle electrode from the small foot muscles. Representative plots from all treatment groups are shown. (F) Sciatic nerve paraffin sections were H&E stained. Scale bar represents 100 μm. (G) The cellular density per section was quantified. n = 10 per group. Pla placebo.

As expected in this chronically progressive model, the average clinical score continuously increased in placebo treated mice (Fig 1A). In comparison to these controls, IVIg 3x treated animals developed a significantly lower disease severity (Fig 1A) after six, eight and nine weeks of treatment. The difference between placebo and IVIg 3x treated mice was most pronounced at the end of the observation (Fig 1B). IVIg 1x treatment generated a similar trend, but the difference did not reach statistical significance (Fig 1A and 1B). We next analyzed the incidence of any clinically detectable impairments. Cumulative neuritis incidence in the placebo group increased to 91% in contrast to 69% in the IVIg 3x group indicating a reduction by IVIg 3x treatment although the survival analysis did not reach significance (Fig 1C). Of note, the response to IVIg treatment was highly heterogenous and clinical scores were negatively correlated with NCV (r = - 0.49, p < 0.001) and CMAP amplitudes (r = - 0.54, p < 0.001) (Fig 1D). Electrophysiological abnormalities were generally more pronounced in placebo than in IVIg treated animals (Fig 1E) but differences did not reach statistical significance (data not shown), which may be due to high technical variability. We next histologically quantified neuritis severity and found that cellular infiltration into the PNS was reduced by IVIg 3x treatment in comparison to placebo (Fig 1F and 1G). Cellular infiltration negatively correlated with NCV and CMAP amplitudes and positively correlated with clinical scores (S1 Fig) argueing for the adequancy of this histological quantification of nerve inflammation. In summary, IVIg 3x treatment thus ameliorates the average severity and suppresses the manifestation of chronic murine neuritis in ICAM-1^-/-^NOD mice.

### Nerve MRI in Mice Allows Quantifying Treatment Effects in ICAM-1^-/-^NOD Mice

**Fig 2:**
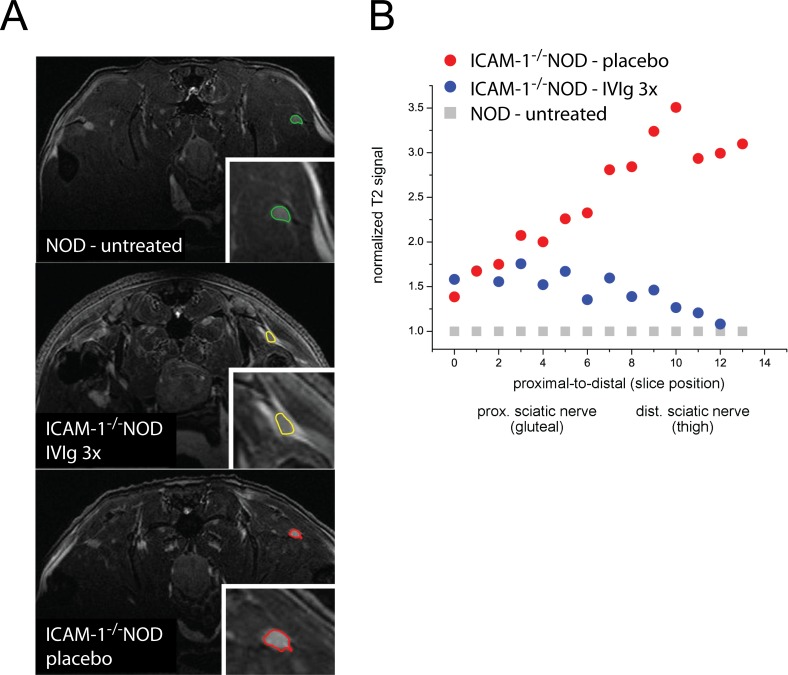
Sciatic nerve MRI detects abnormalities and IVIg effects in ICAM-1^-/-^NOD mice. (A) Untreated, pre-diabetic NOD mice without clinical impairments (n = 3), IVIg 3x treated ICAM-1^-/-^NOD mice (n = 6), and placebo treated ICAM-1^-/-^NOD mice (n = 4) were analyzed in a 9.4T small animal MRI. Anesthesia was performed with 4% isofluran in O_2_ during imaging. A circular polarized cryoprobe was used in Tx/Rx setting and we acquired T2w images. Sciatic nerves were segmented and the T2 signal was normalized to NOD controls using ImageJ. (B) The sciatic nerve was manually outlined on consecutive image slices from proximal to distal anatomical location. The T2 signal was normalized against surrounding muscle tissue and then normalized using NOD nerve intensity as reference. The relative T2 intensity of one representative placebo treated ICAM-1^-/-^NOD mouse (red circles) and one IVIg 3x treated ICAM-1^-/-^NOD mouse (blue circles) was plotted against anatomical position.

Nerve histology is not feasible for longitudinal studies and we intended to establish an alternative, non-invasive quantification of PNS inflammation in mice during treatment. We therefore performed 9.4T sciatic nerve MRI in NOD mice and treated and untreated ICAM-1^-/-^NOD mice. The sciatic nerve could be readily identified from the gluteal to the thigh region on T2 weighted MRI images. We found that T2 intensity was higher in ICAM-1^-/-^NOD mice than in NOD control mice and correlated with the severity of clinical and electrophysiological abnormalities (Fig 2A). We quantified this observation by first normalizing sciatic nerve T2 intensity against the surrounding muscle tissue and second normalizing this relative T2 intensity of ICAM-1^-/-^NOD sciatic nerves against NOD controls.

We then segmented the sciatic nerve into 17 individual sections from proximal (parallel to the first caudal vertebra; 0–8) to distal (parallel to the second caudal vertebra; 9–17) and found that the normalized T2 signal was higher in ICAM-1^-/-^NOD mice over the entire length of coverage. This higher T2 signal in ICAM-1^-/-^NOD nerves was more pronounced in distal parts of the sciatic nerve (Fig 2B). Interestingly, the normalized T2 intensity was unchanged by IVIg treatment in proximal sections whereas IVIg 3x treatment preferentially reduced distal T2 hyperintensities (Fig 2B). To correlate MRI findings with histology we stained for myelin (S2A Fig) and cell content (S2B Fig) at different sites of the sciatic nerve of ICAM-1^-/-^NOD mice. Both demyelination and cellular infiltration correlated well with clinical and electrophysiological parameters (S2 Table) and distal nerve parts were more severely affected. Similar to MRI, histology thus shows a proximal-to-distal gradient of abnormailities in our model. This indicates that nerve MRI is a feasible, non-invasive measure of PNS inflammation and inflammatory demyelination in our murine model of chronic neuritis and that IVIg treatment effects can be monitored by nerve MRI. IVIg may act preferentially in distal parts of the PNS.

### High IL-17 Production in Blood T Cells Predicts Treatment Response to IVIg

**Fig 3:**
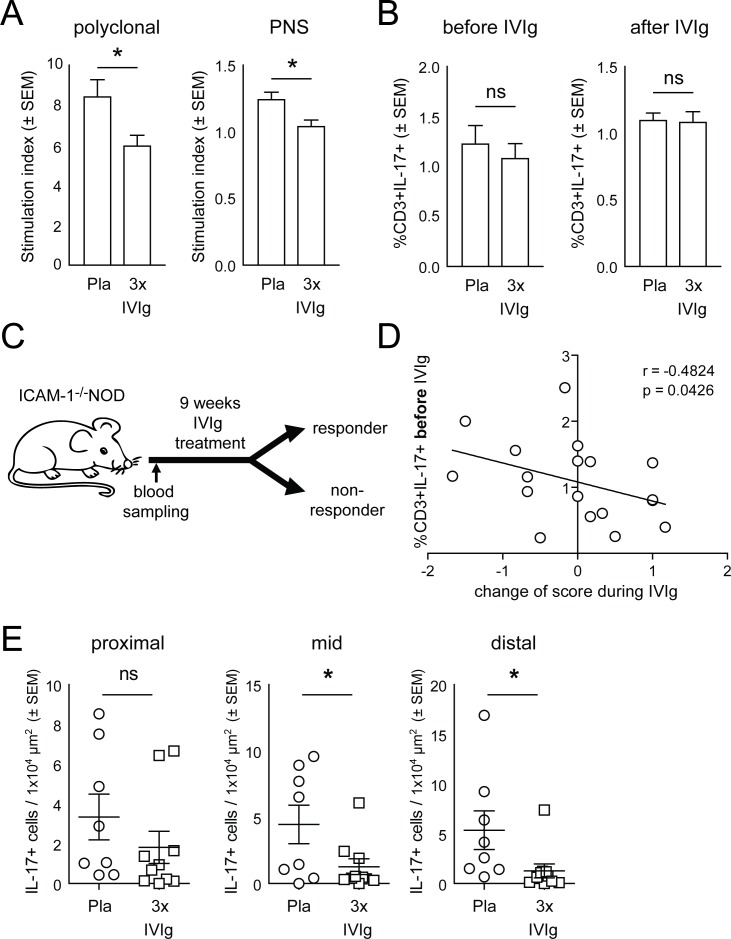
Pre-treatment IL-17 production by blood T cells predicts IVIg response in ICAM-1^-/-^NOD mice. (A) Splenocytes were extracted from placebo (Pla) and IVIg 3x treated mice (n = 5 per group) after 9 weeks of treatment and stimulated in the presence of soluble anti-CD3 (1μg/ml) and anti-CD28 (0.5 μg/ml) antibodies (polyclonal) or mouse sciatic nerve homogenisate (PNS; 50 mg/ml) for 96 hours. Proliferation was measured by Thymidin incorporation in the last 24 hours and calculated as fold non-stimulated wells. (B) Peripheral blood was collected before (left panel) from placebo (Pla) and IVIg 3x treated mice (n = 20 per group), stimulated for 4 hours with PMA/Ionomycin/Golgi Transport Inhibitor and stained for intracellular IL-17. Splenocytes were extracted from treated mice after treatment (right panel) and stained for intracellular IL-17. The proportion of IL-17+ of CD3+ cells before (left panel) and after (right panel) treatment was calculated. (C) Schematic representation of the prognostic analysis. Responders were defined as ≤1 average score increase during treatment. (D) The proportion of IL-17+ CD3+ cells was plotted against the change of score during IVIg 3x treatment. (E) Paraffin sections were generated at different levels of the sciatic nerve at the sciatic notch (proximal, left panel), at mid thigh (mid, middle panel), at the sciatic trifurcation (distal, right panel) were stained for IL-17, IL-17+ cells were counted and the density of IL-17+ cells per area was calculated. ns not significant.

We next tested how IVIg 3x treatment influenced the spontaneous autoimmune response against PNS myelin we had previously identified in ICAM-1^-/-^NOD mice [16]. We found that both polycloncal and myelin induced proliferation of splenic lymphocytes was reduced by IVIg 3x treatment (Fig 3A). We had previously observed development of neuritis in ICAM-1^-/-^NOD mice to be associated with an increased production of pro-inflammatory IL-17 [16]. We therefore performed intracellular cytokine staining of splenic lymphocytes to study the mechanism by which IVIg treatment ameliorated chronic neuritis. Surprisingly, IVIg 3x treatment did not alter IL-17 production by T lymphocytes (Fig 3B) indicating that effects of IVIg treatment in peripheral lymphoid organs are primarily IL-17-independent.

Predicting the individual response to IVIg in CIDP is clinically highly relevant, but no predictive biomarkers have been identified. We therefore next addressed whether our animal model could also be used to identify such prognostic biomarkers by comparing pre-treatment cytokine production with the treatment response (Fig 3C). The percentage of IL-17 producing T cells in the peripheral blood *before* IVIg treatment negatively correlated with the change in clinical score during treatment (Fig 3D) while the percentage of cytokine-producing cells *after* treatment did not correlate (S1B Fig). High levels of IL-17 producing T cells were thus associated with a beneficial response to IVIg in ICAM-1^-/-^NOD mice. This suggests that IL-17 could serve as a predictor of IVIg treatment responses in chronic neuritis.

We next addressed how IVIg affected IL-17 production in the PNS and therefore stained sections of the sciatic nerve at proximal, middle, and distal levels for IL-17. We found that the density of IL-17+ cells was significantly decreased in mice treated with 3x IVIg at middle and distal levels of the sciatic nerve (Fig 3E). This observation indicates that IVIg treatment reduces the production of pro-inflammatory IL-17 in the peripheral nerves of ICAM-1^-/-^NOD mice. Taken together our data suggest that IL-17 production in the blood predicts subsequent treatment responses to IVIg (Fig 3D) and that IVIg does not change IL-17 production in secondary lymphoid organs (Fig 3B), but may decrease IL-17 production locally in the inflamed PNS (Fig 3E).

### IL-17 Production Is Elevated in Sural Nerve Biopsies of Early Stage CIDP Patients

**Fig 4:**
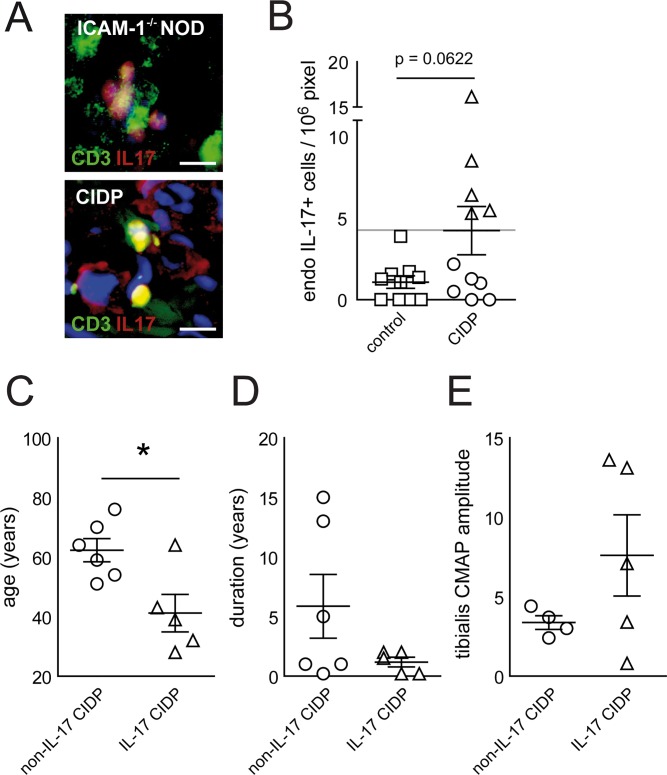
The intraneural presence of IL-17 producing cells dichotomizes CIDP patients. (A) Paraffin sections of sciatic nerves of ICAM-1^-/-^NOD mice (top panel) and representative sural nerve biopsies of CIDP patients (bottom panel) were stained against CD3 (green signal) and IL-17 (red signal) and counterstained with DAPI (blue signal). Scale bars represent 20 μm. (B) The density of endoneural IL-17+ cells in human sural nerve biopsies was compared between controls (squares) and CIDP patients (circles and triangles) by DAB based IL-17 staining. CIDP samples were arbitrarily divided into having high (triangles, IL-17-CIDP) or low (circles, non-IL-17-CIDP) densities of IL-17+ cells. (C-E) Clinical features age (C), disease duration before biopsy (D) and tibial nerve CMAP amplitudes (E) were retrospectively compared between IL-17-CIDP and non-IL-17-CIDP patients.

We next sought to examine the relevance of IL-17 in human CIDP. Data regarding IL-17 producing T cells in CIDP are limited [21–23]. First, we histologically analyzed IL-17 production in the PNS of CIDP patients and found IL-17 producing T cells sural nerve biopsies of CIDP patients similar to sciatic nerves of ICAM-1^-/-^NOD mice (Fig 4A). We therefore asked whether increased IL-17 production in ICAM-1^-/-^NOD mice was paralleled in human chronic inflammatory neuropathies. We took an immunohistochemical approach to study IL-17 production at the site of inflammatory injury in sural nerve biopsy cross-sections of CIDP patients. When we quantified this, we found a statistically marginally non-significant trend (p = 0.0622) towards increased endoneural IL-17+ cells in sural nerve biopsy tissue from CIDP patients compared to non-CIDP controls (Fig 4B, S3 Table). Of note, the presence of IL-17+ cells dichotomized CIDP patients into samples with (IL-17-CIDP) or without (non-IL-17-CIDP) presence of IL-17+ cells in the PNS when we chose an arbitrary threshold defined by our control patients (Fig 4B).

We next compared the clinical and electrophysiological characteristics of IL-17-CIDP and non-IL-17-CIDP patients (S3 Table). We found that IL-17-CIDP patients were significantly younger (Fig 4C), had a trend towards shorter self-reported disease duration (Fig 4D) and higher tibial nerve CMAP amplitudes (Fig 4E) than non-IL-17-CIDP patients, while histological parameters did not differ (S3 Table). This indicates that IL-17 may be involved in the local immune response in the PNS in CIDP. High IL-17 production could thus be associated with high disease activity that is preferentially reduced by IVIg treatment and such patients may thus respond better to IVIg therapy.

## Discussion

We had previously established ICAM-1^-/-^NOD mice as a novel animal model of human CIDP. Here, we used this model to make important discoveries in chronic neuritis. First, IVIg treatment ameliorates chronic neuritis in ICAM-1^-/-^NOD mice and this treatment response features great heterogeneity. This opens up an angle to experimentally study this heterogeneity. Second, nerve MRI is feasible and allows quantifying treatment-dependent nerve abnormalities in a rodent model of chronic nerve inflammation. Nerve MRI may thus also allow tracking treatment effects in chronic inflammatory neuropathies. Third, prospectively quantified IL-17 production by blood T cells predicts the response to IVIg treatment in ICAM-1^-/-^NOD mice and intraneural IL-17 production is enhanced preferentially in early stage human CIDP patients. These findings support the adequacy of the ICAM-1^-/-^NOD model and set the stage for subsequent studies directed at identifying prognostic markers of the highly heterogenous IVIg treatment response in CIDP.

MRI abnormalities correlated with clinical impairments in our model and were most pronounced in distal parts of the sciatic nerve while MRI demonstrated IVIg treatment effects most prominently in distal parts of the sciatic nerve. We thus show that nerve T2 signal is a useful marker to monitor inflammatory neuropathy in mice and can also be used to monitor and localize effects of IVIg treatment.

Peripheral nerve MRI has been used to study many different aspects of peripheral nerve diseases (reviewed in [24]). Recent studies have utilized contrast enhancement agents to visualize different cellular components and aspects of nerve damage and regeneration [25–28]. More recent studies have also used the diffusion tensor imaging (DTI) technique to assess axonal integrity and regeneration by MRI [29].

Several studies have reported T2 signal hyperintensities of the affected peripheral nerve but also reported that MRI signal alterations cannot be directly equated with one type of lesion or a primary cause of disease [30]. It has been speculated that T2 hyperintensities reflect axonal injury and persistent signal abnormailities may even allow to detect inadequate axonal regeneration [24, 31]. T2 hyperintensities may thus reflect axonal degeneration and may in fact even allow assessing axonal regeneration. Acute immune-mediated and chronic inherited demyelination on the other hand did not result in obvious T2 hyperintensities in another study [32]. We therefore speculate that the T2 hyperintensities we observed, reflect ongoing axonal degeneration. In our animal model of CIDP, cellular infiltration, inflammatory demyelination, and axonal degeneration are closely linked [16] because all abnormalities result from T cell autoreactivity against the PNS. The ICAM-1^-/-^NOD mouse is thus poorly suited to dissect the relative contribution of demyelination, axonal loss and cellular infiltration in causing specific MRI alterations. Performing nerve MRI in this model of CIDP, however, does allow monitoring treatment effects and pre-clinically testing and localizing the primariy site of action of such treatments.

Other studies have reported an increase in IL-17 production in CIDP. The percentage of peripheral Th17 cells and IL-17 plasma levels were increased in human CIDP patients with active disease [33]. Concentrations of IL-17 and IL-6, which induces IL-17, were also reported to be increased in the cerebrospinal fluid of CIDP patients [22]. Others reported an increased IL-17 production by blood mononuclear cells in CIDP patients, although this was not specific for this cytokine and IFN-γ levels were also increased [21]. Thus, our findings demonstrating increased intraneural IL-17 production in CIDP are in accordance with previous studies and IL-17 may constitute a future prognostic marker of IVIg treatment response.

IVIg is a preparation of human polyclonal IgG obtained from plasma of several thousands of healthy donors [34] and has demonstrated therapeutic efficacy in CIDP [35]. However, the mechanism of action of IVIg remains incompletely understood and probably cannot be attributed to one specific mechanism of action. Potential mechanisms of IVIg include: anti-idiotype effects, inhibition of the complement pathway, Fc receptor modulation on macrophages and other effector cells, suppression of pathogenic cytokines, and effects on cell migration and direct effect on remyelination (reviewed in [34, 36]). Human IVIg have previously demonstrated efficacy in murine models of inflammatory neuropathies [11, 13] and in murine models of other diseases such as Alzheimer’s disease and rheumatoid arthritis [37, 38]. The IVIg effects are thus at least partly species independent and have been attributed to the Fc portion of the immunoglobulins (reviewed in [39]). This supports our approach of utilizing human IVIg preparations to treat a murine autoimmune disease model. In addition, our study was specifically designed to identify prognostic markers of the IVIg treatment response in CIDP and we identify IL-17 as one potential prognostic factor in chronic inflammatory neuropathies.

Our study has certain technical limitations: Despite stratification by both pre-treatment NCV and clinical score we observed a considerable degree of inter-individual variability in clinical score and response to treatment. The differences between treatment groups were thus not very pronounced. IVIg treatment also did not significantly change electrophysiology measures after treatment (data not shown). Interestingly, electrophysiology measures were also not significantly altered by IVIg treatment in human CIDP patients [35] although electrophysiology correlated with clinical impairments [40]. We speculate that more clearly demonstrating IVIg efficacy may require a longer treatment duration or further increase of IVIg dosage. An alternative approach could be to synchronize the disease onset of chronic neuritis by adoptive transfer into immunodeficient hosts as we have previously reported [41] and treating the recipients with IVIg.

Prospectively collecting samples from early stage human CIDP patients during IVIg treatment is challenging. CIDP diagnosis is often made with considerable delay and due to its cost, IVIg is often used as a second-line treatment. This prognostic aspect can be addressed with relative ease in our model. Compared to human patients, a relatively homogenous cohort of mice can be treated even before the onset of severe clinical impairments. Our attempt to identify a prognostic biomarker of treatment response to IVIg was pre-biased by our previous findings that IL-17 production is enhanced in ICAM-1^-/-^NOD mice. It will be an important future direction of study to perform prospective, un-biased, genome-wide expression analyses in IVIg-treated ICAM-1^-/-^NOD mice.

In conclusion, we here utilized a previously established model of chronic inflammatory neuropathies to identify a potential prognostic biomarker and to establish nerve MRI as a non-invasive tool for treatment monitoring, that sets the stage for future studies.

## Supporting Information

S1 Fig Cellular nerve infiltration correlates with electrophysiological and clinical measures of neuritis. (A) Sciatic nerve electrophysiology was performed and nerve conduction velocity (NCV) and compound muscle action potential (CMAP) amplitudes of the right sciatic nerve were measured after 9 weeks of IVIg treatment. Sciatic nerve paraffin sections were H&E stained and the cellular density per section was quantified. The NCV (top left panel) and CMAP amplitude (top right panel) and the clinical score after treatment (bottom panel) were plotted against the cellular density per sciatic nerve section in each animal. Pearson’s correlation coefficient and significance level are indicated in each panel. (B) Splenocytes were extracted from treated mice after treatment, stimulated for 4 hours with PMA/Ionomycin/Golgi Transport Inhibitor, and stained for intracellular IL-17. The proportion of IL-17+ CD3+ cells in the blood after IVIg was plotted against the change of score during IVIg 3x treatment.
(PDF)

S2 Fig Demyelination and cellular infiltration show a proximal-to-distal increase in ICAM-1^-/-^NOD mice. (A) Sciatic nerve sections from proximal, mid, and distal parts of the sciatic nerve of NOD and ICAM-1^-/-^NOD mice were stained with Luxol Fast Blue (LFB) and the intraneural LFB staining intensity was quantified (0 white– 255 black). (B) Sciatic nerve sections described in A were H&E stained and the cellular density was quantified.
(PDF)

S1 Table Characteristics of randomized ICAM-1^-/-^NOD groups before IVIg treatment. *SD* standard deviation, *NCV* nerve conduction velocity.
(XLS)

S2 Table Clinical, electrophysiological and histopathological features of ICAM-1^-/-^NOD mice analyzed by both MRI and histology in the present study. *LFB* Luxol Fast Blue, *CMAP* compound muscle action potential, *NCV* nerve conduction velocity.
(XLS)

S3 Table Clinical, electrophysiological and histopathological features of CIDP and control patients analyzed in the study. *endo* IL-17 density of endoneural IL-17+ cells (cells per mm^2^), *epi* IL-17 density of epineural IL-17+ cells (cells per mm^2^), *m* male, *f* female, *PNP* polyneuropathy, *HNPP* hereditary neuropathy with liability to pressury palsies, *Lyme* Lyme borreliosis, *sens* sensory symptoms, *dur* duration of disease before sural nerve biopsy (years), *mot* motor symptoms, *recur* recurrent, *progr* progressive, *resp* response, *tib* tibial nerve, *sur* sural nerve, *amp* amplitude, *ncv* nerve conduction velocity, *csf prot* cerebrospinal fluid protein concentration (mg/dl), *csf* cell cerebrospinal fluid cell count (/μl), *ax* axonal, *demy* demyelinating, *T endo* endoneural T cell density (cells per mm^2^), *T epi* epineural T cell density (cells per mm^2^), *av* average, *p* p-value calculated in Student’s t-test for unrelated samples.
(XLS)
