# Supplementary figures and images for: Predicting the Response to Intravenous Immunoglobulins in an Animal Model of Chronic Neuritis
Source: PLoS One. 2016 Oct 6;11(10):e0164099. doi: 10.1371/journal.pone.0164099 (PMC5053527; doi:10.1371/journal.pone.0164099)

# S1 Figure

A

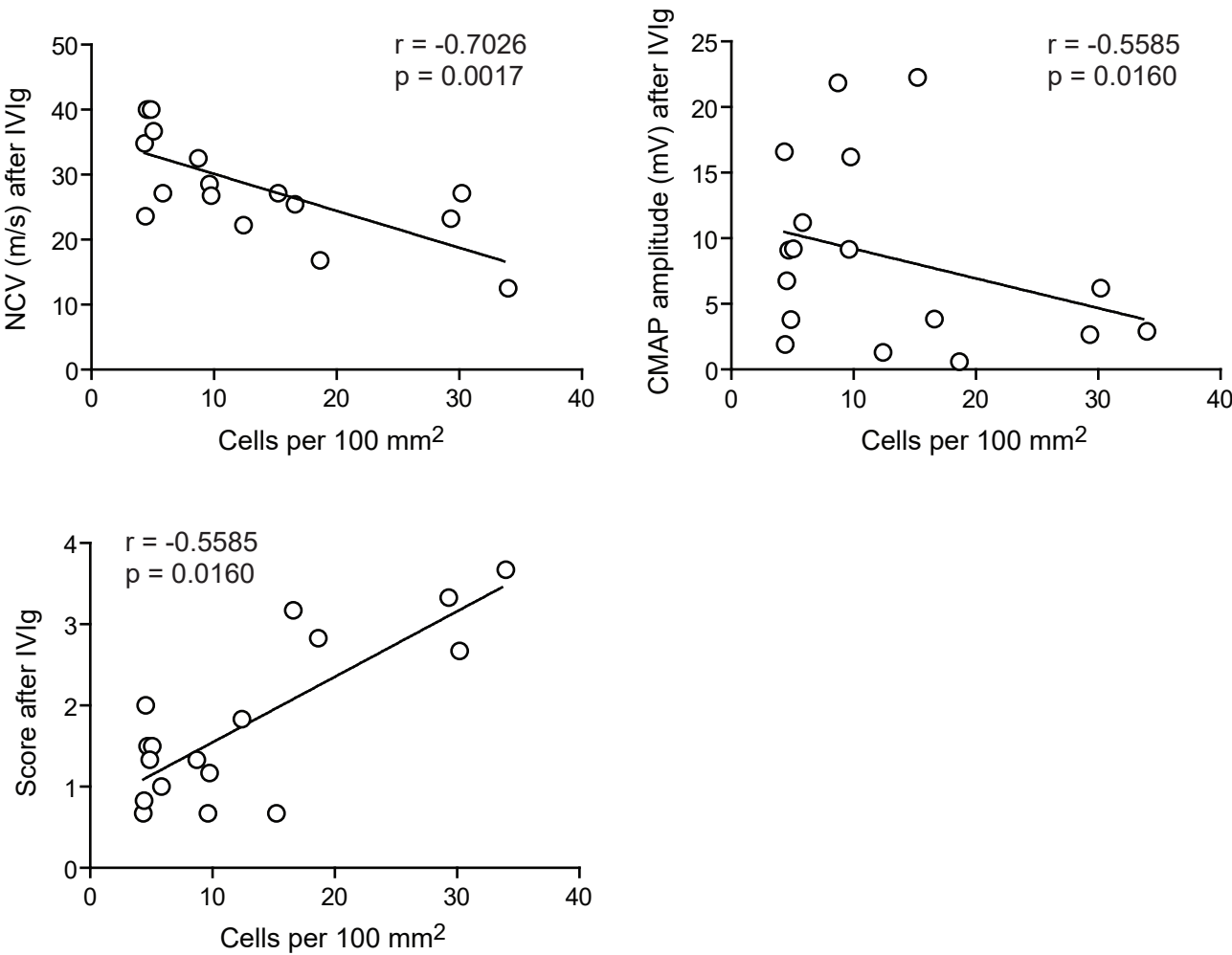

B

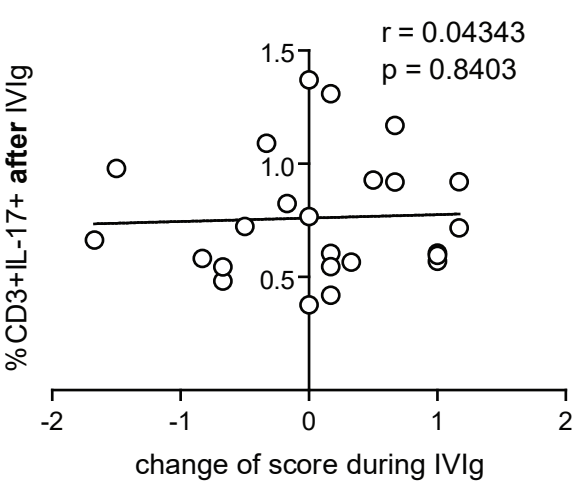

Supplement: S1 Fig — (A) Sciatic nerve electrophysiology was performed and nerve conduction velocity (NCV) and compound muscle action potential (CMAP) amplitudes of the right sciatic nerve were measured after 9 weeks of IVIg treatment. Sciatic nerve paraffin sections were H&E stained and the cellular density per section was quantified. The NCV (top left panel) and CMAP amplitude (top right panel) and the clinical score after treatment (bottom panel) were plotted against the cellular density per sciatic nerve section in each animal. Pearson’s correlation coefficient and significance level are indicated in each panel. (B) Splenocytes were extracted from treated mice after treatment, stimulated for 4 hours with PMA/Ionomycin/Golgi Transport Inhibitor, and stained for intracellular IL-17. The proportion of IL-17+ CD3+ cells in the blood after IVIg was plotted against the change of score during IVIg 3x treatment. (PDF) [file pone.0164099.s001.pdf]

S2 Figure

A

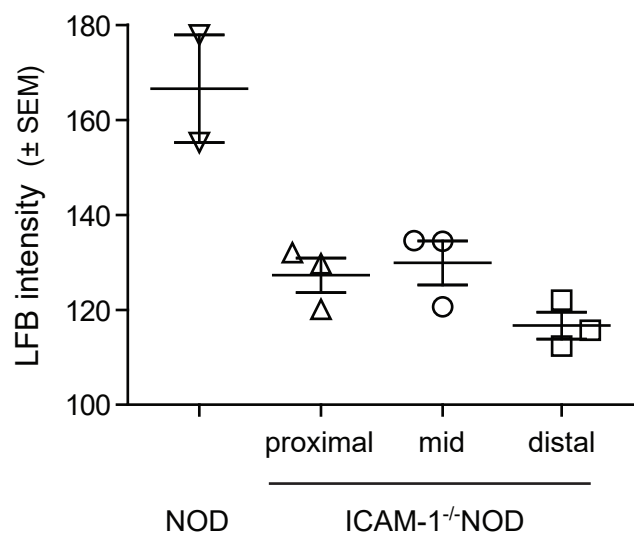

B

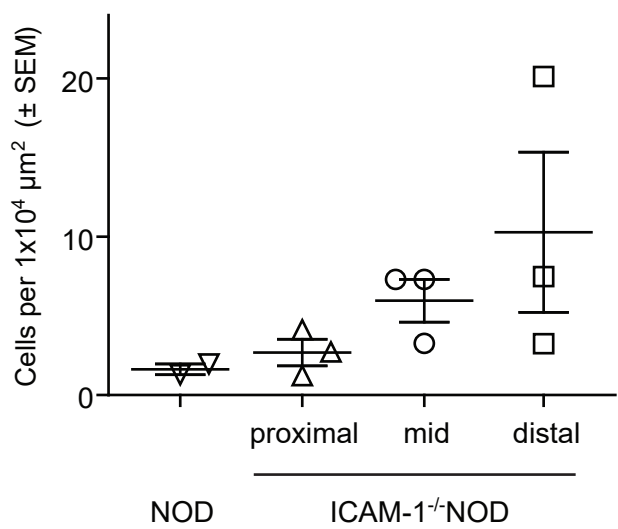

Supplement: S2 Fig — (A) Sciatic nerve sections from proximal, mid, and distal parts of the sciatic nerve of NOD and ICAM-1-/-NOD mice were stained with Luxol Fast Blue (LFB) and the intraneural LFB staining intensity was quantified (0 white– 255 black). (B) Sciatic nerve sections described in A were H&E stained and the cellular density was quantified. (PDF) [file pone.0164099.s002.pdf]
